# Identification of key genes in rheumatoid arthritis and osteoarthritis based on bioinformatics analysis

**DOI:** 10.1097/MD.0000000000010997

**Published:** 2018-06-01

**Authors:** Naiqiang Zhu, Jingyi Hou, Yuanhao Wu, Geng Li, Jinxin Liu, GuiYun Ma, Bin Chen, Youxin Song

**Affiliations:** a Second Department of Spinal Surgery, the Affiliated Hospital of Chengde Medical College; b Hebei Key Laboratory of Study and Exploitation of Chinese Medicine, Chengde Medical College; c Key Lab of Chinese Medicine Resources Conservation, State Administration of Traditional Chinese Medicine of the People's Republic of China, Institute of Medicinal Plant Development, Chinese Academy of Medical Sciences and Peking Union Medical College, Beijing; d Department of Rheumatology and Immunology, First Teaching Hospital of Tianjin University of Traditional Chinese Medicine, Tianjin; e China-Japan Friendship Hospital, Beijing, China.

**Keywords:** differentially expressed genes, osteoarthritis, protein-protein interaction network, regulatory network, rheumatoid arthritis, underlying pathogenesis

## Abstract

Rheumatoid arthritis (RA) and osteoarthritis (OA) comprise the most common forms of arthritis. The aim of this study was to identify differentially expressed genes (DEGs) and associated biological processes between RA and OA using a bioinformatics approach to elucidate their potential pathogenesis.

The gene expression profiles of the GSE55457 datasets, originally produced through use of the high-throughput Affymetrix Human Genome U133A Array, were downloaded from the Gene Expression Omnibus (GEO) database. The GSE55457 dataset contains information from 33 samples, including 10 normal control (NC) samples, 13 RA samples, and 10 OA samples. Gene ontology (GO) and Kyoto Encyclopedia of Genes and Genomes pathway (KEGG) enrichment analyses were performed to identify functional categories and associated molecular and biochemical pathways, respectively, for the identified DEGs, and a protein-protein interaction (PPI) network of the DEGs was constructed using Cytoscape software.

GO and KEGG results suggested that several biological pathways (ie, “immune response,” “inflammation,” and “osteoclast differentiation”) are commonly involved in the development of both RA and OA, whereas several other pathways (eg, “MAPK signaling pathway,” and “ECM-receptor interaction”) presented significant differences between these disorders.

This study provides further insights into the underlying pathogenesis of RA and OA, which may facilitate the diagnosis and treatment of these diseases.

## Abstract 2

Supplemental Digital Content is available in the text

## 1 Introduction

Rheumatiod arthritis (RA) is a complex, chronic multi-systemic autoimmune disease that mainly affects the flexibile joints.^[1]^ Approximately 0.5% to 1% of the adult population is affected with RA worldwide 5 to 50 per 100,000 new cases reported annually.^[2]^ Although disease etiology remains unknown, the progressive, chronically inflammatory, and destructive joint characteristics of RA are perpetuated by an invasive inflammatory tissue also known as pannus tissue.^[3]^ In addition, various inflammatory cells in the synovial membrane (including monocytes/macrophages, T and B cells, osteoclasts, dendritic cells, endothelial cells, and synovial fibroblasts) contribute to the development and progression of RA.^[4]^ Similarly, osteoarthritis (OA) constitutes another main type of common and chronic disease that affects the joints. OA mainly affects individuals older than with a reported disease incidence as high as 80%.^[1]^ OA is also a progressive disorder that is characterized by degradation of the cartilage and bone, along with osteophyte formation, narrowing of the joint space and subchondral sclerosis.^[5]^ Although RA and OA share similar symptoms, OA differs from RA in that these diseases exhibit different pathological manifestations for the synovial. The characteristics of RA are synovial cell hypertrophy and hyperplasia along with infiltration of lymphocytes and inflammatory cells, whereas OA has a fewer leukocytic infiltrates.^[6,7]^ Despite significant advances toward an understanding of the pathophysiology of RA and OA, early diagnosis, therapeutic intervention, and underlying pathogenesis remain a challenging.^[8]^ Therefore, elucidating the unique characteristics belonging to RA or OA is paramount in developing therapies to improve patient outcome.

Recently, numerous research strategies have explored the molecular characteristics of RA and OA. Among these research strategies, high-throughput microarray methodologies have received extensive attention and engendered substantial progress in field such as medical oncology with marked clinical applications ranging from molecular diagnosis to molecular classification, patient stratification to prognosis prediction, and new drug targets discovery to response prediction.^[9–11]^ In addition, multiple gene expression profiling studies on OA and RA have been performed using microarray technology and several key genes and diagnostic biomarkers have been identified for these diseases, including the profiling of hundreds of differentially expressed genes (DEGs) involved in different pathways, biological processes, or molecular functions.^[12,13]^ However, owing to the underlying shortcomings of microarray technology, such as small sample size, measurement error, and information not sufficient, the integrative analysis of multiple factors that contribute to the development of RA and OA has proven to be a major challenge, and underlying pathogenesis of RA and OA thus remaining poorly understood.

Therefore, in the present study, we downloaded the original data (GSE55457), provided by Dirk Woetzel, from the publically available Gene Expression Omnibus database (GEO, http://www.ncbi.nlm.nih.gov/geo/) to identify DEGs and the associated biological processes between OA and RA using comprehensive bioinformatics analyses. The DEGs were subjected to functional enrichment and pathway analyses; moreover, a protein-protein interaction (PPI) network was constructed to screen for key gene nodes. Analysis of the biological functions and pathways shared between and unique to these disorders may shed light on further insights regarding OA and RA development at the molecular level and pave the way toward understanding potential disease pathogenesis mechanisms to facilitate diagnosis, prognosis, and the identification of drug targets.

## 2 Methods

### 2.1 Data resources

Gene expression profile dataset GSE55457^[14]^ was downloaded from the GEO database (http://www.ncbi.nlm.nih.gov/geo/). The data was produced using an Affymetrix Human Genome U133A Array (Agilent Technologies, Santa Clara, CA). The GSE55457 dataset contained data from 33 samples, including 10 NC, 13 RA, and 10 OA samples.

### 2.2 Data acquisition and preprocessing

The original datasets including soft-formatted family files, serial Matrix files, and Robust Multi-array Averaging (RMA) tar files, were downloaded for further analysis. The analysis was carried out using GeneSpring software (version 11.5, Agilent). Arrays from the Affymetrix platform were preprocessed by robust multi-array. We used a classical *t* test to identify DEGs with *P* < .05 and fold change ≥2 as being statistically significant. Ethical approval was not necessary in our study because the expression profiles were downloaded from the public database and no new experiments in patients or animals were performed.

### 2.3 Gene ontology (GO) and pathway enrichment analysis of DEGs

The GO analysis^[15]^ database (http://geneontology.org/), a set of a large number of gene annotation terms, can be used to annotate genes and gene products and identify characteristic biological attributes for high-throughput genome or transcriptome data.^[15,16]^ The Kyoto Encyclopedia of Genes and Genomes (KEGG)^[17]^ knowledge database (http://www.kegg.jp/) is a knowledge base for the systematic analysis of gene functions, linking genomic information with higher-order functional information.^[18]^ The Database for Annotation, Visualization, and Integrated Discovery (DAVID) (http://david.ncifcrf.gov/)^[19]^ constitutes an essential online tool for data synthesis that serves as a foundation for successful high-throughput gene functional analyses.^[20]^ In this study, the identified DEGs were subjected to GO enrichment and KEGG pathway analysis using DAVID. *P* < .05 was used as the cutoff criterion for the functional enrichment analysis.

### 2.4 Integration of the PPI network and analysis of key genes therein

The Search Tool for the Retrieval of Interacting Genes/Proteins (STRING: http://string-db.org/) is online biological database and website designed to evaluate PPI information.^[21]^ Proteins associated with DEGs (species: *Homo*) were selected based on information in the STRING database (PPI score >0.7), and then PPI networks were constructed using Cytoscape software (http://cytoscape.org/).^[22]^ In this study, degree centrality, which constitutes a fundamental parameter in network theory, was adopted to evaluate the nodes in a network. Degree centrality is defined as the number of adjacent links; that is, the number of interactions that connect one protein to its neighbors. The degree centrality method was calculated using Cytoscape plugin CytoHubba.

### 2.5 Module analysis

The plug-in Molecular Complex Detection (MCODE)^[23]^ is used to detect densely connected regions in PPI networks. In this study, we selected significant modules from the constructed PPI network using MCODE. The criteria settings were as follows: node score cutoff = 0.2, K-Core = 2, and degree cutoff = 2. The MCODE score was then calculated. In addition, the functional and pathway enrichment analyses of DEGs in each module were performed using DAVID, with a *P* < .05 used as the cutoff criterion for the functional enrichment analysis.

## 3 Results

### 3.1 Identification of DEGs

A total of 313 genes were identified to be differentially expressed between RA and NC samples with the threshold of *P* < .05 and a minimal 2-fold change of expression. Among these DEGs, 101 were up-regulated and 212 down-regulated in RA compared with NC samples (Supplementary list 1). The top 10 up- and down-regulated genes for RA and NC are list in Table 1. Similarly, 208 DEGs were identified to be differentially expressed between OA and NC samples, including 150 up-regulated and 58 down-regulated genes (Supplementary list 2). In addition, a total of 160 DEGs between RA and OA were identified, including 82 up-regulated and 78 down-regulated genes (Supplementary list 3). The top 10 DEGs for OA vs. NC and RA vs. OA samples are listed in Table 2 and Table 3, respectively.

**Table 1:**
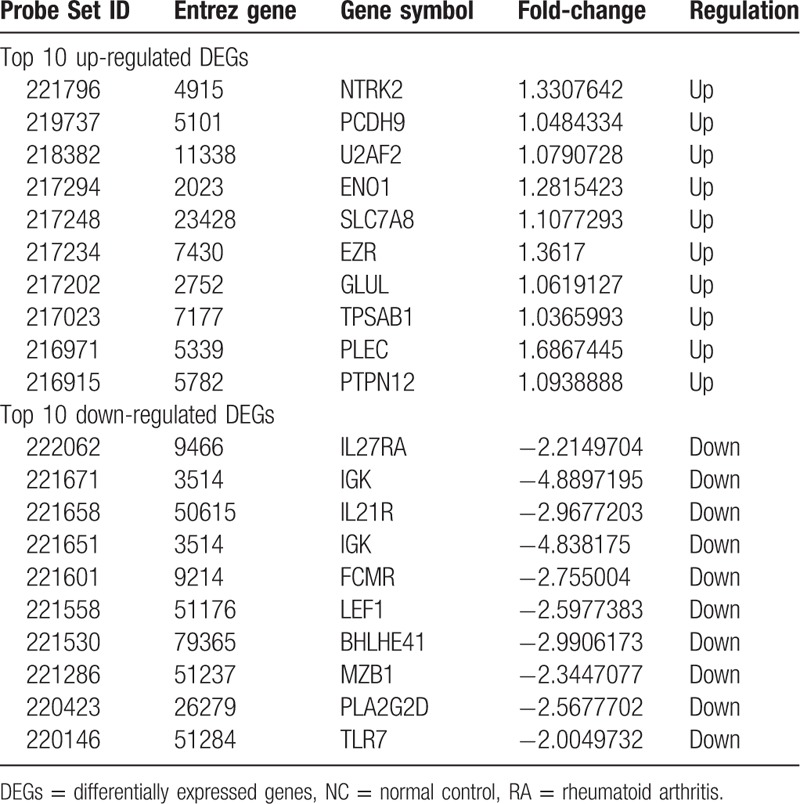
The top 10 up- and down-regulated DEGs in RA and NC.

**Table 2:**
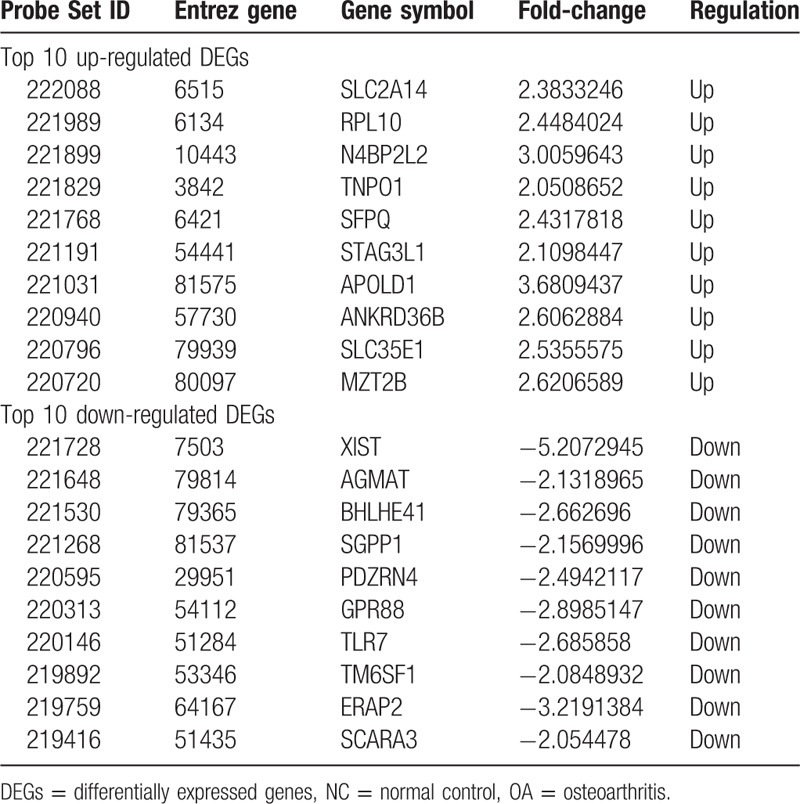
The top 10 up- and down-regulated DEGs in OA and NC.

**Table 3:**
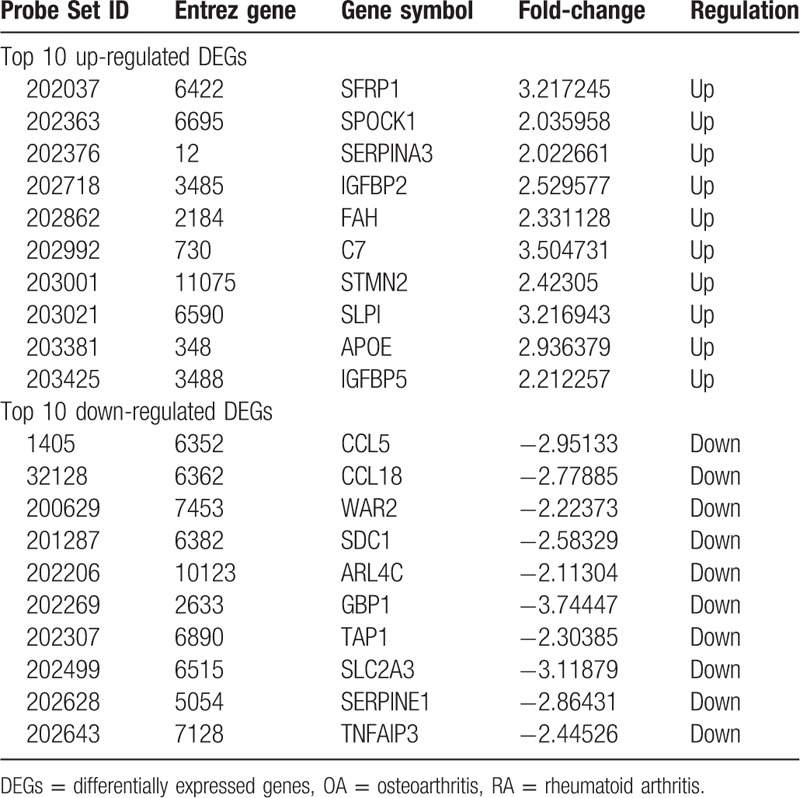
The top 10 up- and down-regulated DEGs in RA and OA.

### 3.2 Enrichment analysis of DEGs

**Figure 1:**
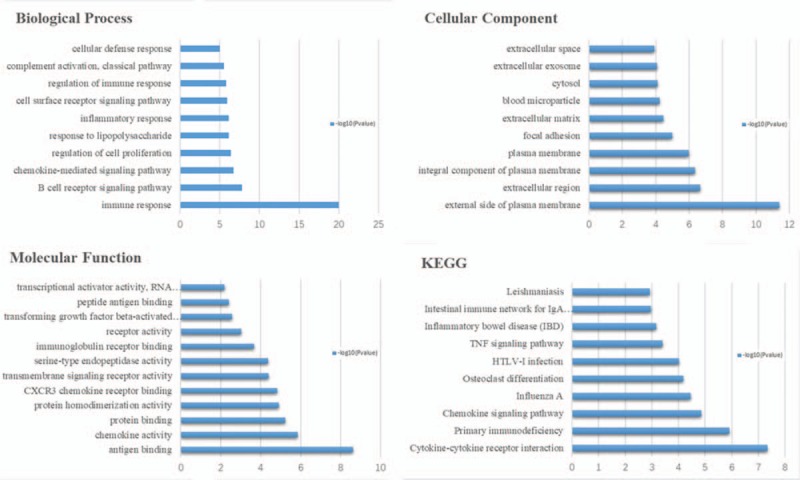
The biological process, cellular component ,and molecular function terms, and KEGG pathways enriched by the DEGs between RA and NC. DEGs = differentially expressed genes, KEGG = Kyoto Encyclopedia of Genes and Genomes, NC = normal control, RA = rheumatoid arthritis.

**Figure 2:**
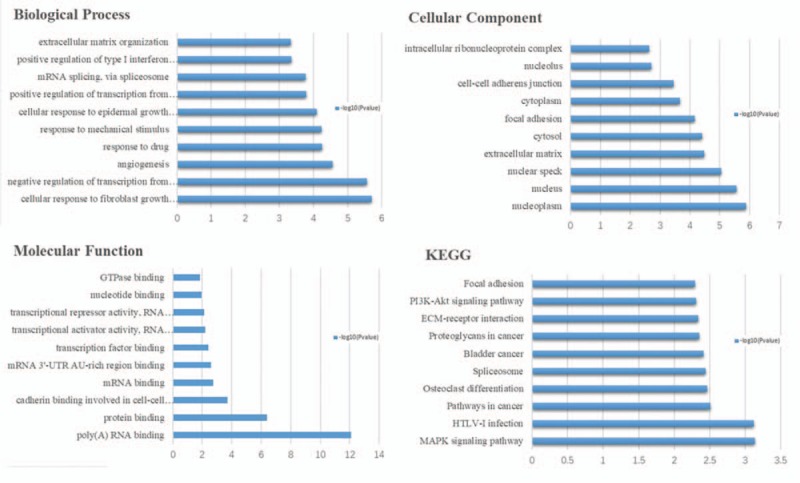
The biological process, cellular component and molecular function terms, and KEGG pathways enriched by the DEGs between OA and NC. DEGs = differentially expressed genes, KEGG = Kyoto Encyclopedia of Genes and Genomes, NC = normal control, OA = osteoarthritis.

**Figure 3:**
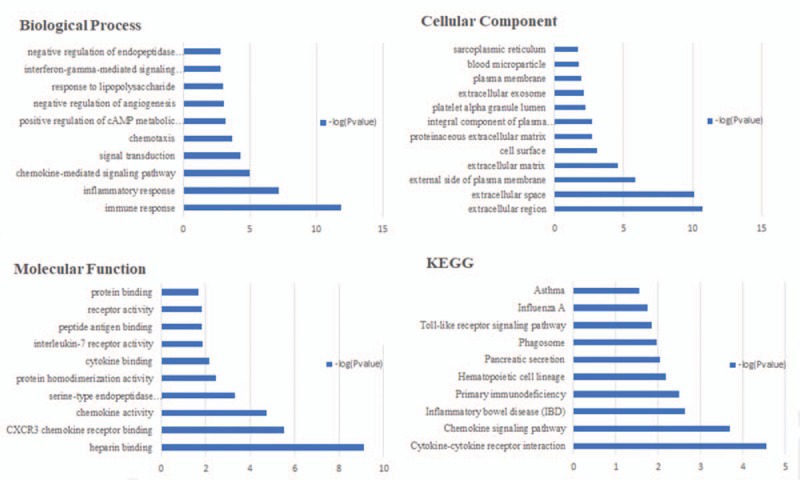
The biological process, cellular component and molecular function terms, and KEGG pathways enriched by the DEGs between RA and OA. DEGs = differentially expressed genes, KEGG = Kyoto Encyclopedia of Genes and Genomes, OA = osteoarthritis, RA = rheumatoid arthritis.

To gain insights into the biological roles of the DEGs from RA and OA versus NC samples, we performed GO categories enrichment analysis. In this study, all DEGs were uploaded to the online software DAVID in order to identify overrepresented GO categories. GO term enrichment analysis results varied according to GO classification and expression change of DEGs. With the criterion of *P* < .05, “immune response” (*P* = 1.7E- 20), “response to drug” (*P* = 2.3E- 6), and “inflammatory response” (*P* = 3.6E- 11) exhibited highly significant enrichment within the GO biological process category. For the cellular component category, DEGs were significantly enriched in “external side of plasma membrane” (*P* = 5.3E-12), “nucleoplasm” (*P* = 3.9E- 5), and “extracellular space” (*P* = 1.4E-11). In addition, the molecular function category contained DEGs significantly enriched in “antigen binding” (*P* = 2.9E-9), “poly(A) RNA binding” (*P* = 3.0E-9), and “heparin binding” (*P* = 5.6E-9). KEGG pathway enrichment analysis was then used to understand the signaling pathway enrichment of DEGs. With the criterion of *P* < .05, the top enriched biological pathways associated with RA and OA included “cytokine-cytokine receptor interaction”, “mitogen-activated protein kinase (MAPK) signaling pathway,” “chemokine signaling pathway,” “human T-lymphotropic virus 1 infection,” and “extracellular matrix (ECM)-receptor interaction” (Figs. 1–3).

### 3.3 PPI network analysis of DEGs

To further explore the relationships between DEGs at the protein level, the PPI networks were constructed based on the interactions of DEGs. With the predefined criterion of combined score > 0.7, a total of 3605 interactions and 797 nodes were screened to establish the PPI network between RA and NC samples (Supplementary Fig. 1). In this network, the top 15 key genes with highest degree scores are shown in Table 3. Similarly, the PPI networks regarding OA versus NC and RA versus OA samples were constructed including 696 nodes, 2991 edges and 466 nodes, 1923 edges, respectively (Supplementary Figs. 2–3). Among these nodes, the top 15 hub genes with the highest degree scores are shown in Table 4.

**Table 4:**
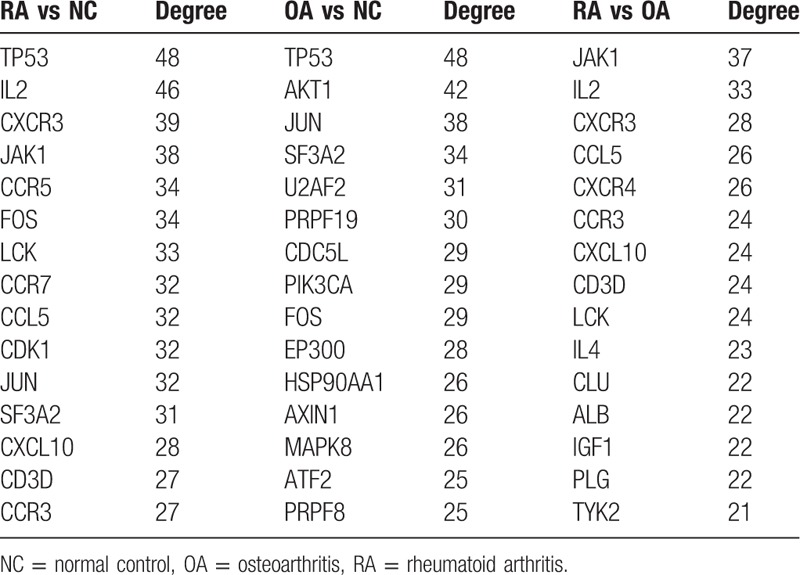
The top 15 hub genes with highest degree scores.

### 3.4 Module analysis

**Figure 4:**
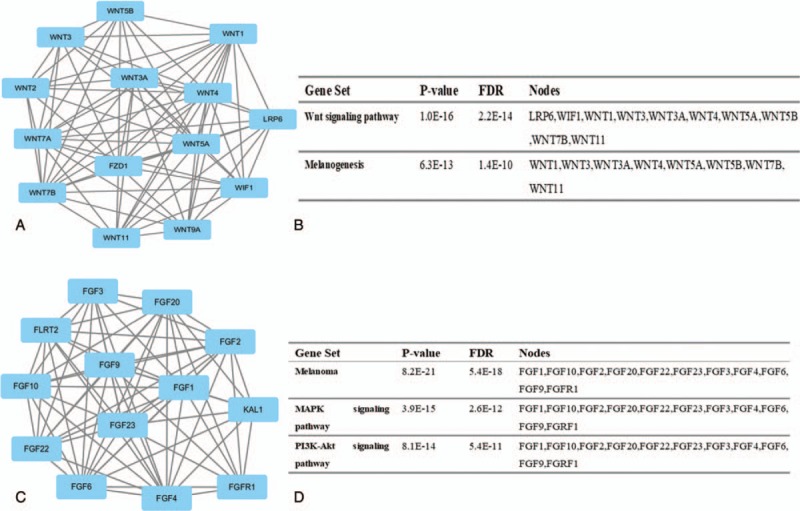
Top 2 modules from the protein-protein interaction network between RA and NC: (A) module 1, (B) the KEGG enriched pathways of module 1, (C) module 2, (D) the KEGG enriched pathway of module. KEGG = Kyoto Encyclopedia of Genes and Genomes, NC = normal control, RA = rheumatoid arthritis.

**Figure 5:**
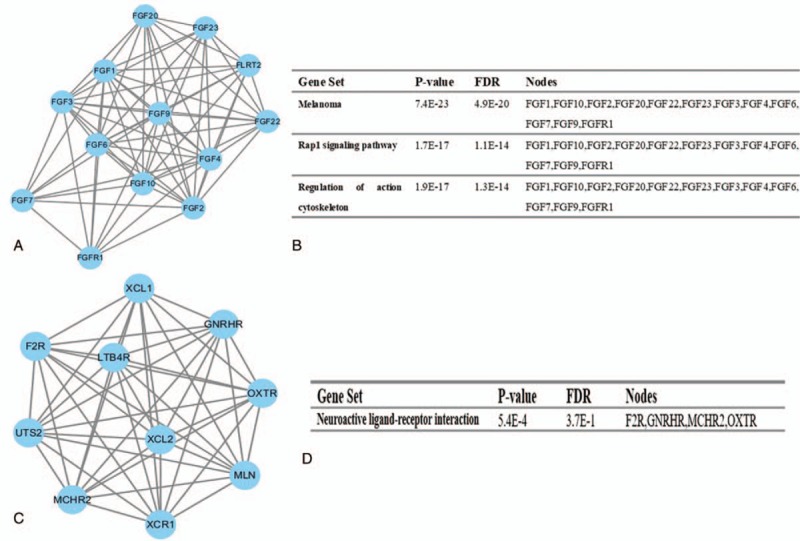
Top 2 modules from the protein-protein interaction network between OA and NC: (A) module 1, (B) the KEGG enriched pathways of module 1, (C) module 2, (D) the KEGG enriched pathway of module 2. KEGG = Kyoto Encyclopedia of Genes and Genomes, NC = normal control, OA = osteoarthritis.

**Figure 6:**
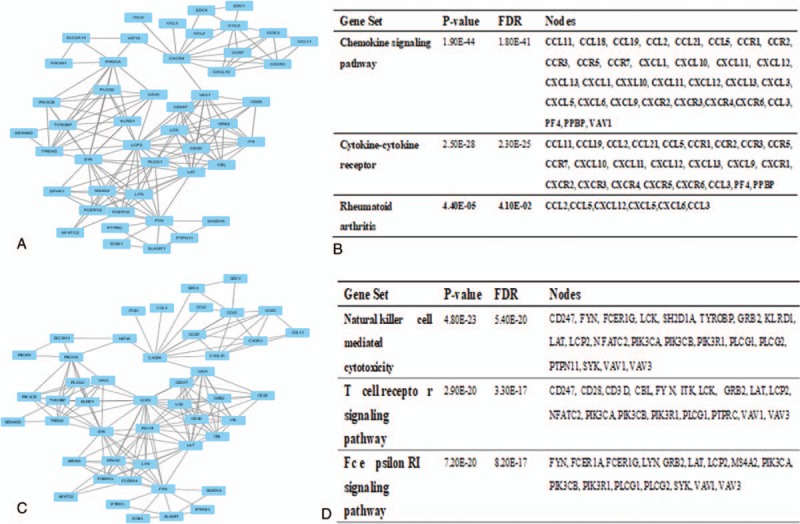
Top 2 modules from the protein-protein interaction network between RA and OA: (A) module 1, (B) the KEGG enriched pathways of module 1, (C) module 3, (D) the KEGG enriched pathway of module. KEGG = Kyoto Encyclopedia of Genes and Genomes, OA = osteoarthritis, RA = rheumatoid arthritis.

The top 2 significant modules of RA versus NC, OA versus NC, as well as RA versus OA samples were selected, respectively, and functional annotation of the genes involved in the modules was analyzed (Figs. 4–6). KEGG enrichment analysis showed that the genes of RA versus NC samples in modules 1 to 2 and OA versus NC samples were mainly associated with “melanoma,” “neuroactive ligand-receptor interaction,” “Rap1 signaling pathway,” “Wnt singaling pathway,” and “MAPK signaling pathway.” Furthermore, the DEGs of RA versus OA were significantly clustered in “chemokine signaling pathway” and “Jak-STAT signaling pathway.”

## 4 Discussion

RA and OA, the most common forms of arthritis, constitute chronic painful conditions that affect an individual's quality of life.^[24]^ However, the potential causes of RA and OA remain uncertain. Understanding the underlying pathogenesis of RA and OA is of critical importance for diagnosis, prognosis, and identifying drug targets. As high-throughput sequencing and microarray technology can provide information regarding the expression levels of thousands of genes in the human genome simultaneously, this methodology has been widely used to predict the potential diagnostic and therapeutic targets for OA and RA. In the present study, we extracted the data from GSE55457, which includes 10 NC samples, 13 RA samples, and 10 OA samples. We identified 101 up-regulated and 212 down-regulated DEGs between RA and NC samples using bioinformatics analysis. As cumulative evidence has shown that co-expressed genes normally represent those with similar expression profiles that also frequently participate in similar biological processes,^[25]^ to better understand the interactions of the identified DEGs, we further performed GO and KEGG functional annotation and pathway enrichment analyses. These analyses suggested that the identified DEGs were mainly involved in “immune response,” “chemokine-mediated signaling pathway,” “B cell receptor signaling pathway,” “integral component of plasma membrane,” “antigen binding,” “cytokine-cytokine receptor interaction,” “osteoclast differentiation,” and “tumor necrosis factor (TNF) signaling pathway.”

In addition, a total of 208 DEGs including 150 up- and 58 down-regulated DEGs were identified between OA and NC samples. The majority of DEGs were enriched in a limited number of functional categories, such as “negative regulation of transcription from RNA polymerase promoter,” “nucleoplasm, focal adhesion,” “osteoclast differentiation,” “EMC-receptor interaction,” “MAPK signaling pathway,” and “phosphatidylinositol 3-kinase-protein kinase B (PI3K-Akt) signaling pathway.” Moreover, 160 DEGs were identified between RA and OA samples, including 82 up- and 78 down-regulated DEGs. In this case, significantly enriched functions included “negative regulation of endopeptidase,” “sarcoplasmic reticulum,” “protein binding,” and “blood microparticle.” Overall, the top enriched biological pathways for the both RA and OA included “chemokine signaling pathway,” “osteoclast differentiation,” “MAPK signaling pathway,” and “focal adhesion.” Furthermore, by constructing PPI networks, we identified some key genes that provide new insights for RA and OA diagnosis, prognosis, and drug target identification.

Chemokines comprises a family of small cytokines or proteins secreted by immune cells that regulate and control cells of the immune system during the processes of immune response.^[1]^ Accumulating evidence suggested that chemokines can become involved at RA development and progression.^[26]^ In the present study, DEGs involved in the chemokine signaling pathway were identified to be altered in both RA and OA, including the gene clusters chemokine (C-C motif) ligand 13 (*CCL13*), *CCL18*, *CCL5*, chemokine (C-C motif) receptor 2 (*CCR2*), *CCR5*, chemokine (C-X-C motif) ligand 10 (*CXCL10*), *CXCL11, CXCL13, CXCL9*, interleukin 2-inducible T-cell kinase, chemokine (C motif) ligand 1, neutrophil cytosolic factor 1, protein kinase, cAMP-dependent, catalytic, beta, and signal transducer, and activator of transcription 1. Specifically, these chemokine-related DEGs were down-regulated in RA samples. Therefore, increased expression of these genes in RA might not have a potential role for inflammation activation in the pathogenesis of RA.

Osteoclasts derived from multinucleated giant cells are responsible for remodeling and resorption of bone^[27,28]^ comprise specialized monocyte/macrophage family members that differentiate from hematopoietic precursors that appear to stem from a common monocyte precursor. The process of osteoclast differentiation from precursor cells involves the expression of proteins that together identify cells of the osteoclast lineage, including cell surface receptors (calcitonin receptors, c-fms), lysosomal enzymes (TRAP), cathepsin K, intracellular signaling molecules (c-src), and transcription factors (PU.1). Numerous studies have shown that osteoclast differentiation plays an important role in RA bone destruction.^[29,30]^ In the present study, several DEGs of RA were enriched in osteoclast differentiation, such as FBJ murine osteosarcoma viral oncogene homolog, transcription factor family JUN (*JUN)*, Jun B proto-oncogene, interleukin 1 receptor type 1 (*IL1R1*), suppressor of cytokine signaling 3, and transforming growth factor, beta receptor 2 (*TGFBR2*). As these were up-regulated in the RA samples, increased expression of these genes in RA might constitute a potential therapeutic target of RA.

The MAPK family includes a serious of serine/threonine protein kinases that are involved in regulating numerous cell functions, such as differentiation, proliferation, apoptosis, and inflammatory responses.^[31]^ In comparison, the involvement of MAPK in the regulation of the inflammatory response and the development and progression of RA has been widely identified.^[32]^ MAPK pathway inhibition can be regarded as an anti-inflammatory therapy, thus suggesting these DEGs as potential targets of anti-inflammatory therapy for OA and RA.^[33]^ In the present study, DEGs that were found to be associated with the MAPK signaling pathway included *JUN*, dual specificity phosphatase 1 (*DUSP1*), *DUSP5*, growth arrest and DNA-damage-inducible 45 beta, *IL1R1*, nuclear factor of kappa light polypeptide gene enhancer in B-cells 2, nuclear receptor subfamily 4, group A, member 1, TGFBR2, and myelocytomatosis oncogene, which were up-regulated in the OA samples. Therefore, the increased expression of these genes in OA might indicate potential mechanisms of inflammation activation in OA pathogenesis.

An increasing number of studies suggested that members of focal adhesion kinase (FAK) associated with cell adhesion, cell proliferation, cell migration, and osteoclast differentiation.^[34]^ In this study, we identified several DEGs of OA samples as being enriched in the focal adhesion pathway, including *JUN*, cartilage oligomeric matrix protein, catenin (cadherin-associated protein), beta 1, collagen type IV, alpha 2, collagen type VI alpha 1 placental growth factor, and vascular endothelial growth factor A, which were up-regulated in the OA samples. Therefore, the increased expression of these genes in OA might also constitute a therapeutic and diagnostic target.

The subsequent construction of the PPI network using the respective DEGs identified 15 DEGs as potential key genes involved in OA and RA. Tumor protein 53 *(TP53)* was identified as one of the key genes exhibiting the highest degree of differential expression in both RA and OA samples. *TP53*, also known as the tumor suppressor gene p53, induces cell growth arrest or apoptosis depending on the physiological circumstances and cell type. Tak et al^[35]^ reported that the development of mutations in *TP53* and other key regulatory genes could help convert inflammation into chronic disease in RA and other inflammatory disorders. In addition, Zhu et al^[36]^ reported that the expression of the *TP53* gene is correlated with disease grades of OA. In turn, *IL2*, the second key gene of RA, is crucial to regulation of the immune response, which can stimulate B-cells, monocytes, lymphokine-activated killer cells, natural killer cells, and glioma cells. Notably, recent studies have proposed that *IL2* is associated with the development and progression of RA.^[37]^ The protein encoded by Akt murine thymoma viral oncogene homolog 1 (*AKT1*), the second key gene of OA, is activated by phosphorylation at Thr308 and Ser473. Activated *AKT1* promotes cell growth, survival pathways, and inactivates various components of apoptotic cascade.^[38]^ Accumulating evidence has demonstrated that AKT1 appears to be constitutively activated with increases in both native and phosphorylated forms in OA tissues.^[39]^

Furthermore, module analysis of the PPI network revealed that the development of RA was associated with the PI3K-Akt signaling pathway. This pathway is crucial to many aspects of cell growth and survival in both physiological as well as in pathological conditions.^[40]^ It has been demonstrated that the PI3K-Akt signaling pathway can be activated by several cytokines such as TNF-α in RA synoviocytes.^[41]^ Moreover, module analysis of the PPI network of OA suggested that the development and progression of OA were related to the Wnt signaling pathway. Recent studies have suggested that the Wnt signaling pathway is involved in cartilage distortion and subchondral bone changes.^[42]^ Moreover, the increased expression of Wnt-induced signaling protein 1 has been found in both experimental and human OA.^[43]^

## 5 Conclusions

In conclusion, *TP53, IL2*, and *AKT1*may be key genes for RA and OA. Furthermore, the results of the present study suggested that several biological pathways (ie, “the immune response,” “inflammation,” and “osteoclast differentiation”) are commonly involved in the development of both RA and OA. In comparison, several other pathways (ie, “MAPK signaling pathway,” “ECM-receptor interaction”) presented significant differences between RA and OA. This study thus provides further insights into the underlying pathogenesis of OA and RA, which may facilitate the diagnosis and treatment of these diseases.

## Author contributions

**Conceptualization:** Naiqiang Zhu, Jinxin Liu.

**Methodology:** Naiqiang Zhu.

**Visualization:** Jinxin Liu.

**Writing – original draft:** Naiqiang Zhu, Jinxin Liu.

**Writing – review & editing:** Jingyi Hou, Guiyun Ma, Yuanhao Wu, Geng Li, Bin Chen, Youxin Song.

## Supplementary Material

Supplemental Digital Content

## Supplementary Material

Supplemental Digital Content

## Supplementary Material

Supplemental Digital Content

## Supplementary Material

Supplemental Digital Content
