# Supplementary material for: Identification of key genes in rheumatoid arthritis and osteoarthritis based on bioinformatics analysis
Source: Medicine (Baltimore). 2018 Jun 1;97(22):e10997. doi: 10.1097/MD.0000000000010997 (PMC6392928; doi:10.1097/MD.0000000000010997)

Supplementary Figure 1 Protein-protein interaction networks between RA vs. NC samples

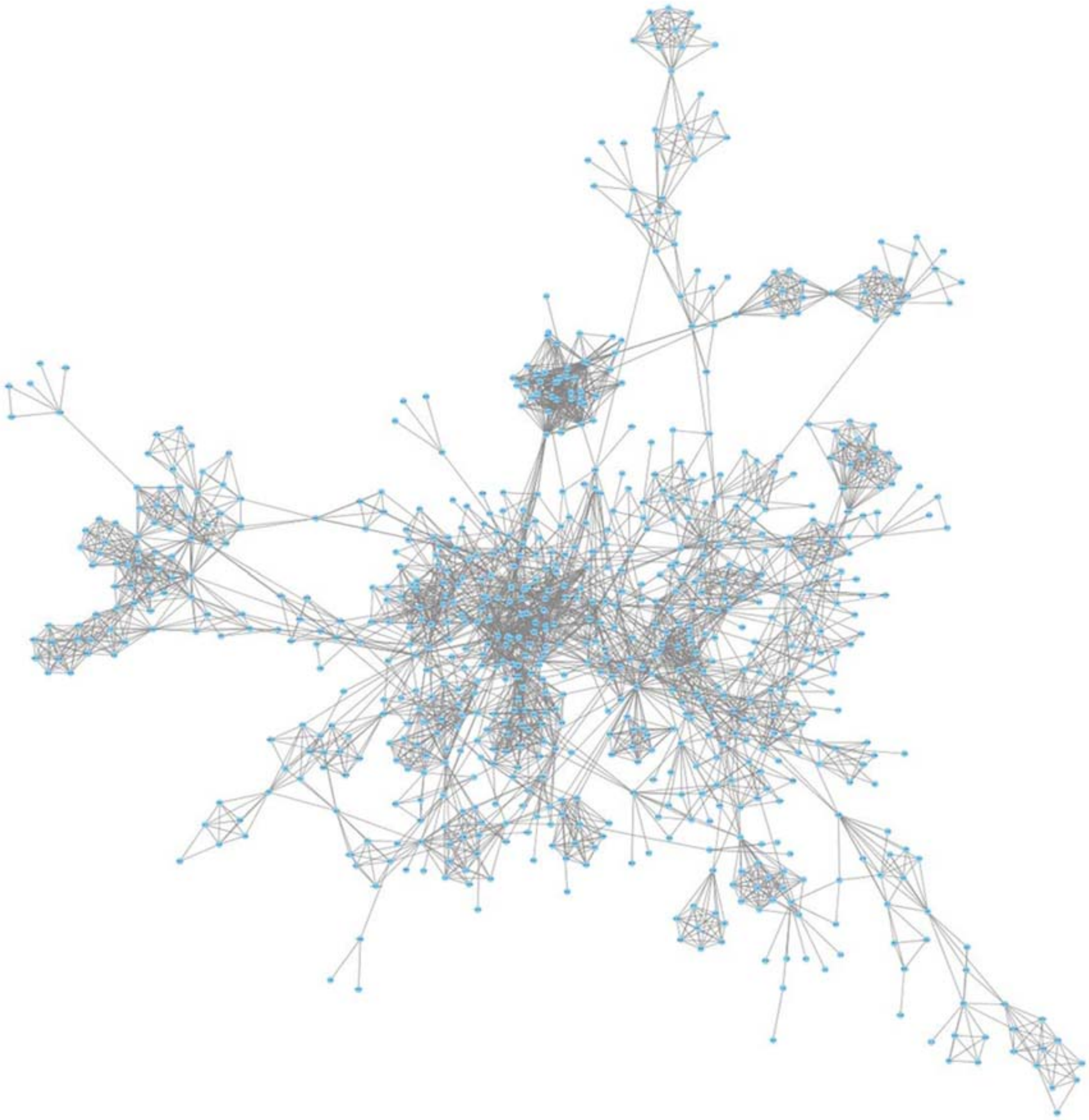

Supplementary Figure 2 Protein-protein interaction networks between OA vs. NC samples

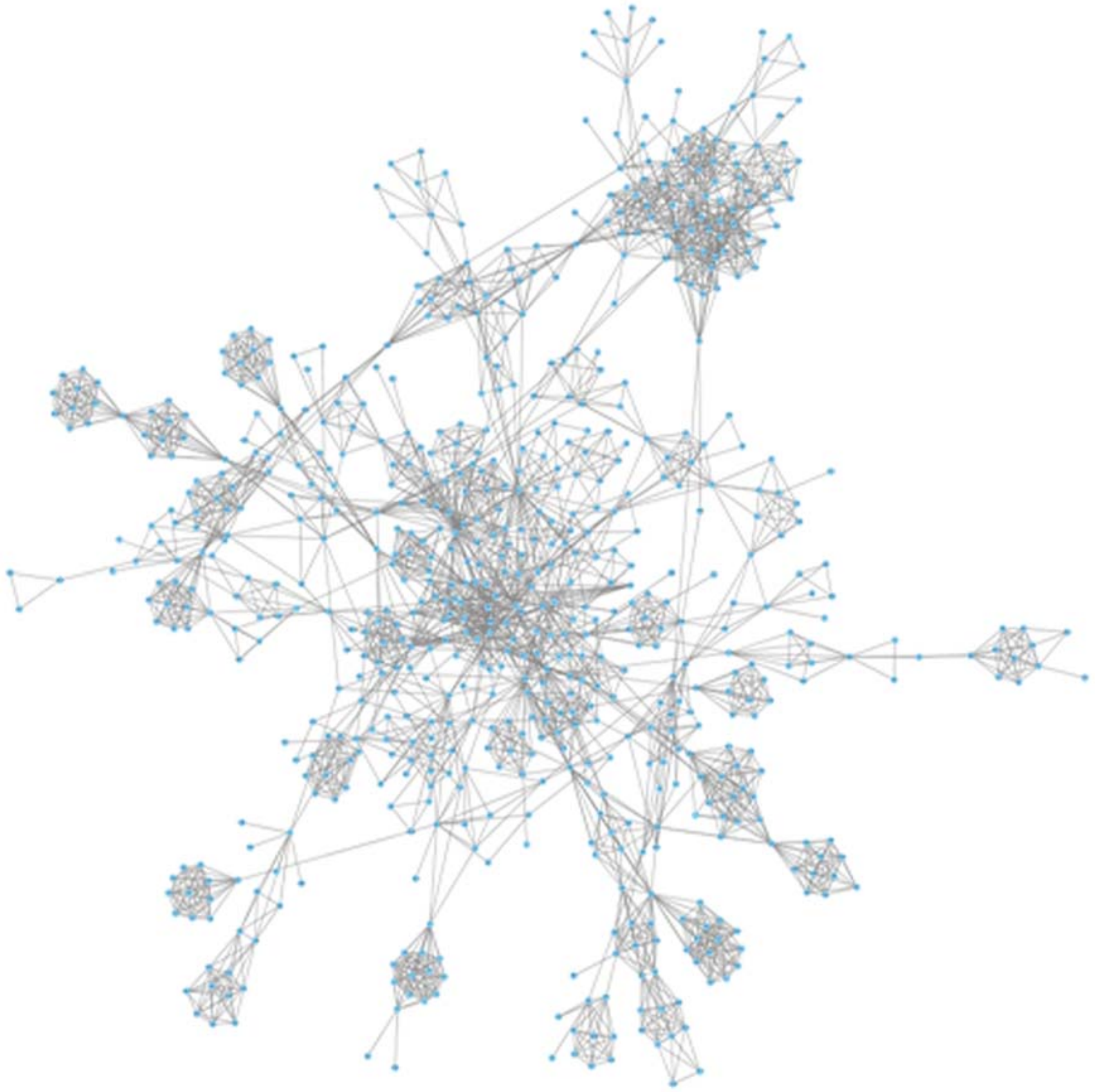

Supplementary Figure 3 Protein-protein interaction networks between RA vs. OA samples

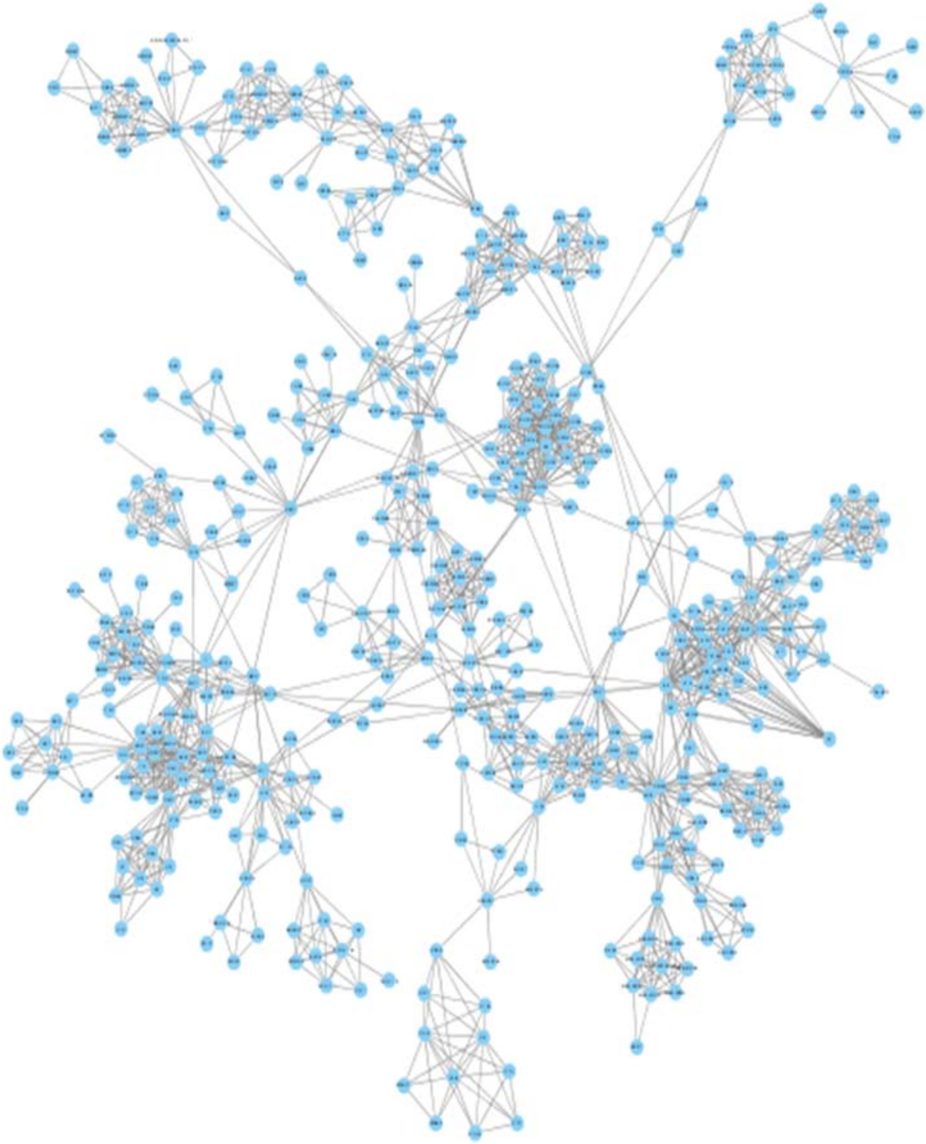

Supplement: Supplemental Digital Content [file medi-97-e10997-s004.pdf]
